# Clinical applications of fucoidan in translational medicine for adjuvant cancer therapy

**DOI:** 10.1186/s40169-019-0234-9

**Published:** 2019-05-01

**Authors:** Hsien-Yeh Hsu, Pai-An Hwang

**Affiliations:** 1 Institute of Taiwan Fucoidan Development, 1F, No. 123-1, Sec. 4, Bade Rd., Songshan Dist., Taipei, 105 Taiwan; 2 Department of Biotechnology and Laboratory Science in Medicine, Institute of Biotechnology in Medicine, National Yang-Ming University, 155 Li-Nong Street, Shih-Pai, Taipei, Taiwan; 3 Department of Bioscience and Biotechnology, National Taiwan Ocean University, Keelung City, Taiwan

**Keywords:** Fucoidan, Polysaccharide, Cancer, Signal transduction, Alternative medicine, Combination treatment, Clinical trials, Anti-cancer chemotherapy, Complementary therapy

## Abstract

The chemical composition of fucoidan, a kind of sulfated polysaccharide mainly derived from brown seaweed, includes a substantial percentage of l-fucose. Fucoidan has various biological and pharmacological activities, such as anti-cancer/anti-tumor, anti-proliferation, anti-inflammatory and immune-modulatory functions, and fucoidan-related dietary supplements and nutraceuticals have recently drawn considerable attention. In this review, we aim to provide a current view of different aspects of fucoidan biological activity, with a focus on the anti-cancer regulatory effects of fucoidan on growth signaling mechanisms. First, we discuss historical aspects of fucoidan and fucoidan products, as well as the anti-cancer effects of fucoidan on various cancer cells. Second, we discuss fucoidan’s biological activities and induction of cell death in cancer cells, including multiple mechanisms and signal transduction pathways related to its anti-cancer effects. Next, we focus on fucoidan and fucoidan-derived products that have been marketed as dietary supplements or nutraceuticals for cancer, including anti-cancer effects of fucoidan when combined as an adjuvant with clinical drugs. Finally, case studies of fucoidan in complementary therapy and as an alternative medicine in animal and mouse models and human clinical trials to alleviate side effects of anti-cancer chemotherapy are discussed. Combining fucoidan with clinical therapeutic agents in the treatment of cancer patients, dissecting the related signal transduction pathways and investigating their dynamic interactions may reveal potential molecular targets in cancer prevention, therapies and key obstacles in the current development of anti-cancer strategies.

## Background

### A brief history of fucoidan and fucoidan products

Fucoidan was first isolated and discovered in 1913 by Dr. Kylin of Uppsala University, Sweden, from brown algae: *Ascophyllum nodosum*, *Fucus vesiculosus*, *Laminaria digitata* and *Laminaria saccharina* [1]. Fucoidan belongs to a large family of marine sulfated polysaccharides named fucans mainly constituted of sulfated l-fucose. These polysaccharides include ascophyllans (xylofucoglycuronan and xylofucomanuronan) and sargassans (glycuronofucogalactan) [2, 3]. Fucoidan, a sticky member of the class of sulfated, fucose-rich polysaccharides is found mainly in the fibrillar cell walls and intercellular spaces of brown seaweeds of the class *Phaeophyceae*, such as kelp, kombu (*Laminaria japonica*), mozuku (*Nemacystus decipiens*, or Mozuku seaweed: Sunui, *Cladosiphon novae*-*caledoniae* Kylin), sargassum (the Atlantic Ocean’s Sargasso Sea is named after the algae), wakame (*Undaria pinnatifida*), hijiki (*Hizikia fusiforme*), nori (*Porphyra tenera*), limumoui, bladderwrack, and another 70 species of brown algae [4–7]. In addition, fucoidan also occurs in the body wall of certain marine invertebrates such as sea cucumber (*Holothuroidea*) and in the egg jelly coat of sea urchins (*Echinoidea*) [8–10].

**Fig. 1:**
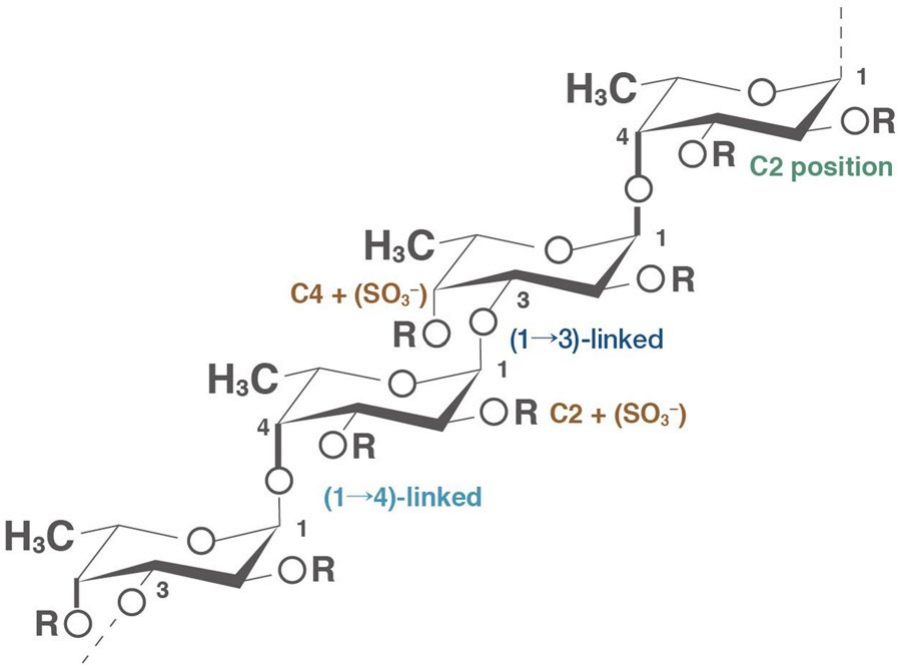
Structure of fucoidan

Structurally, fucoidan has a simple chemical composition and is a heparin-like molecule with an α-1,3-backbone or a repeat unit consisting of disaccharides containing an α-1,3-linked fucose and an α-1,4-linked fucose with branches attached at the C2 positions as shown in Fig. 1. In general, fucoidan contains a substantial percentage of l-fucose polymerized with sulfated ester groups and a small proportion of d-galactose, d-glucose, d-mannose, d-xylose and glucuronic acid residues [2–4, 11], as well as protein, calcium, copper, magnesium, manganese, potassium, selenium, sodium, zinc, and other minerals. In a broad chemical sense, fucoidan refers to polysaccharides mainly composed of sulfated fucose.

Fucoidan, a water-soluble dietary fiber, has various pharmacological and biological activities, such as anti-cancer, anti-inflammatory, and immunomodulatory functions [2, 4, 12], as will be discussed in later sections. Given its advantages of low toxicity and oral bioavailability, fucoidan and fucoidan-derived products have recently been marketed as dietary supplements or nutraceuticals for various diseases, including cancer.

### Anti-cancer effect of fucoidan

Cancer imposes a huge burden on society and has been identified as the leading cause of death in both more and less economically developed countries [13]. In 2012, the International Agency for Research on Cancer (IARC) estimated that there were approximately 14 million new cancer cases and 8.2 million cancer deaths worldwide. By 2030, considering the growth and aging of the world population, 22 million new cancer cases and 13 million cancer deaths are expected annually. Due to changes in lifestyle such as smoking habits, poor diet, physical inactivity, and fewer childbirths, the future cancer burden will probably be even worse in economically developing countries.

Human cancer is characterized by aberrant growth and metastatic abnormal cells; uncontrolled spread (metastasis) results in death of the host. Despite tremendous efforts and progress in medical research, cancer remains one of the major causes of human death worldwide. A battery of therapeutic strategies such as chemotherapy, radiation therapy, surgery and their combinations is used to treat different types of cancer [14]. Unfortunately, several of these treatments provide only minimal benefits; moreover, complications and long-term side effects of these treatments can occur [15, 16]. The therapeutic potential of natural bioactive compounds such as polysaccharides, including fucoidan, is now well-documented. The anti-cancer activity associated with the natural biodiversity of fucoidan from brown seaweeds has supported the development of a new generation of therapeutic measures against cancer over the years [2, 4, 6, 17]; see the discussion sections below.

Numerous studies support the anti-cancer activities of fucoidan in vitro and in vivo, and effects of fucoidan on various cancers in humans have been proposed and demonstrated, here we summarize the results in Table 1. In brief, the anti-cancer effects of fucoidan have been evaluated in cancers such as acute leukemia [18], lymphoma [19, 20], head and neck cancer [21], nasopharyngeal carcinoma [22], oral cancer (or mucoepidermoid carcinoma) [23], esophageal cancer [24], lung cancer [25–27], breast cancer [28–30], gastric cancer [31], hepatoblastoma [32], hepatocellular carcinoma and hepatic cells [33], cholangiocarcinoma [32], prostate cancer [34], colorectal carcinoma [35, 36], renal cancer [32], ovarian cancer [32], cervical cancer [37], bladder cancer [38], and gallbladder cancer [32], among others.

**Table 1:** The anti-cancer effect of fucoidan from brown seaweeds on its cancer target and inhibitory mechanism/reaction

Cancer type	Research method	Fucoidan source	Anti-cancer mechanism/reaction	References
Acute leukemia	In vitro	*Fucus vesiculosus*	Induction of apoptosis	[18]
Lymphoma	In vitro and in vivo	*Fucus vesiculosus*	Oral administration of fucoidan inhibited tumor growth	[19, 20]
Head and neck cancer	In vitro and in vivo	*Fucus vesiculosus*	Injection of fucoidan-based nanoparticles inhibited tumor growth	[21]
Nasopharyngeal carcinoma	In vitro and in vivo	*Laminaria japonica*	Injection of fucoidan inhibited tumor growth and induced apoptosis	[22]
Oral cancer	In vitro	*Fucus vesiculosus*	Caspase-dependent apoptosis	[23]
Lung cancer	In vitro	*Fucus vesiculosus*	Inhibition of tumor migration and invasion	[24]
	In vitro	*Turbinaria conoides*	Induction of apoptosis	[27]
	In vitro and in vivo	*Fucus vesiculosus* * Laminaria japonica*	Oral administration of fucoidan modulated the TGFR/Smad7/Smurf2-dependent axis, leading to TGFR protein degradation and inhibition of lung cancer cell progression	[26]
	*Human*	*Laminaria japonica*	Survival rates of the lung cancer patients using cisplatin and fucoidan (oral administration) were increased by approximately 50% compared with those of the patients using cisplatin alone	[25]
Breast cancer	In vitro and in vivo	*Fucus vesiculosus* * Laminaria japonica*	Injection of fucoidan decreased tumor cell metastasis by enhancing ubiquitin-dependent TGF-β receptor degradation	[28]
	In vitro	*Fucus vesiculosus*	Higher branching degree of fucoidan had greater cytotoxicity of tumor cell	[29]
Gastric cancer	In vitro	*Fucus vesiculosus*	Induction of apoptosis and autophagy	[31]
Hepatoblastoma	In vitro	*Cladosiphon okamuranus tokida*	Induction of apoptosis and inhibition of cell cycle	[32]
Hepatocellular carcinoma	In vitro	*Fucus vesiculosus*	Induction of apoptosis	[33]
Cholangiocarcinoma	In vitro	*Cladosiphon okamuranus tokida*	Induction of apoptosis and inhibition of cell cycle	[32]
Prostate cancer		*Undaria pinnatifida*	Injection of fucoidan decreased tumor volume and increased apoptosis	[34]
Colorectal carcinoma	In vitro	*Saccharina cichorioides*	Low molecular weight fucoidan exhibited more anti-proliferation than native fucoidan	[35]
	In vitro	*Fucus vesiculosus*	Induction of apoptosis and decreased angiogenesis	[36]
Renal cancer	In vitro	*Cladosiphon okamuranus tokida*	Induction of apoptosis and inhibition of cell cycle	[32]
Ovarian cancer	In vitro	*Cladosiphon okamuranus tokida*	Induction of apoptosis and inhibition of cell cycle	[32]
Cervical cancer	*Human*	*Nemacystis decipiens*	Clinical improvement in cancer patients through integrated medicine, mainly using low molecular weight fucoidan supplements	[37]
Bladder cancer	In vitro	*Fucus vesiculosus*	Induction of G1 arrest of the cell cycle through down-regulation of pRB phosphorylation	[38]
Gallbladder cancer	In vitro	*Cladosiphon okamuranus tokida*	Induction of apoptosis and inhibition of cell cycle	[32]

### Fucoidan biological activity and mechanism of induction of cell death in cancer cells

There have provided great and impactful insights on the signaling pathways and mechanisms involved in the anti-tumor activity of fucoidan in cancer. The molecular mechanisms of fucoidan-mediated anti-tumor signal transduction pathways have recently been examined and proposed. In general, fucoidan affects cancer cells via various biological processes or reactions. One biological process/reaction of fucoidan-mediated anti-tumor function may involve single or multiple signaling pathways. The cellular and molecular biology aspects of fucoidan anti-tumor function include fucoidan-induced/mediated (1) apoptosis and anti-proliferation, reactive oxygen species, and endoplasmic reticulum stress; (2) anti-inflammation; (3) cell cycle arrest; (4) anti-angiogenesis; (5) inhibition of metastasis, migration and invasion; and (6) immunological reactions. The anti-cancer activities of fucoidan are likely mediated via multiple signal transduction pathways.

#### 1. Apoptosis and anti-proliferation

Fucoidan induces cell death by promoting apoptosis and/or anti-proliferation in many cancer cells. For example, fucoidan (from *F. vesiculosus*) inhibits HepG2 cell viability and induces apoptosis [33]. *Turbinaria conoides*-derived fucoidan inhibits the growth of A549 cancer cells [39], shows antioxidant activities against various free radicals in A549 cells, and decreases matrix metalloprotease-2 and -9 activities in pancreatic cancer cells [40]. Fucoidan (from *Sargassum cristaefolium*) shows antioxidant activity and inhibition of growth in HT-29 human colon cancer cells [41]. Fucoidan (from *Sargassum horneri*) inhibits the growth of human colon cancer DLD cells [42]. Fucoidan (from *Alaria angusta*) inhibits colony formation in HT-29 and T-47D cell lines [43]. Fucoidan and synthesized low-molecular-weight fucoidan derivatives (type I and II) show apoptosis-inducing activities through activation of caspase-8 and -9 in human breast cancer MCF-7 cells and human cervical epithelioid carcinoma HeLa cells [44]. Fucoidan from *Cladosiphon okamuranus* (Okinawa Mozuku) inhibits human gastric adenocarcinoma cell line MKN45 proliferation by suppressing the ASK1 (apoptosis signal-regulating kinase)-p38 signaling pathway through reduction of phosphorylated ASK1 levels [45].

In general, apoptosis and/or anti-proliferation are major strategies for eradicating various cancers [46]. Apoptosis is one of the most extensively studied forms of programmed cell death and plays a critical role during various physiological processes, including fucoidan-mediated cell death [20]. Caspases play central roles in the mechanism of apoptosis, and there are several pathways by which caspases can be activated, including in fucoidan-induced apoptosis [47]. There are two common types of apoptosis signaling pathways, the mitochondrial pathway (also known as the intrinsic pathway) includes the expression of pro- and anti-apoptotic proteins of the Bcl-2 family, such as Bax, Bid, Bak, Bcl-xL, and Mcl-1 inside cells [46], release of cytochrome c from the mitochondria to the cytosol and subsequent activation of caspase-9, -3 or other caspases. The cell death receptor-mediated pathway (DRP, also known as the extrinsic pathway) includes activation of the NF-κB, PI3K/Akt and MAPK pathways. The MAPK family, such as ERK, JNK and p38, can activate NF-κB and result in cell survival. A third initiation pathway, the intrinsic endoplasmic reticulum (ER) pathway, has been proposed [48]. The signaling pathway of ER stress may be also coupled to two cascades, namely PERK/P-eIF2a/CHOP [48] and ATF6 (IRE-1)/XBP-1. These observations suggest that fucoidan-mediated ER stress can mediate both the extrinsic pathway and intrinsic pathway of apoptosis; for a further discussion, see below.

##### Reactive oxygen species (ROS) and ER stress in fucoidan-mediated cancer cell death

The endoplasmic reticulum (ER) is an important intracellular organelle with many bio-functions, such as protein folding, initial post-translational modification, lipid biogenesis, and maintenance of calcium (Ca^2+^) homeostasis within cells [49]. Induction of ER stress may result in a series of intracellular cell death and apoptosis-related signaling pathways [50]. The effect and mechanism of fucoidan-induced apoptosis via ER stress are unclear. Fucoidan increases intracellular reactive oxygen species (ROS), which are responsible for the increases in ATF4 and CHOP in lung cancer cells. The ROS scavenger *N*-acetyl-l-cysteine (NAC) abolishes fucoidan-induced ROS and inhibits fucoidan-induced ER stress [48]. These results indicate that ROS generation is involved in fucoidan-induced ER stress-mediated apoptosis of lung cancer cells.

Reactive oxygen species are a group of oxygen-containing reactive chemical species. There are two types of ROS: free radicals and non-free radicals [51]. Non-free radical ROS do not have unpaired electrons but can be converted to radical ROS [52]. The inter-relationship among fucoidan, ER stress, ROS and apoptosis in cancer cells has been studied. A fucoidan extract of Mozuku seaweed (*C. novae*-*caledoniae* Kylin) activates a caspase-independent apoptotic pathway in human breast cancer MCF-7 cells through ROS-dependent JNK activation and mitochondrial-mediated Bcl-2 family pathways [53]. Fucoidan (*U. pinnatifida* sporophylls) induces apoptosis in human hepatocellular carcinoma SMMC-7721 cells via the ROS-mediated mitochondrial pathway [54]. Fucoidan (*F. vesiculosus*) exerts its anti-tumor function and induces apoptosis of human MDA-MB-231 breast cancer cells and HCT116 colon cancer cells by modulating ER stress cascades [55]. Fucoidan also inhibits the proliferation of the human myelodysplastic syndrome (MDS)/acute myeloid leukemia (AML) cell line SKM-1 via the activation of apoptotic pathways and ROS production [56]. Induction of unbalanced ROS in cancer cells via therapeutic approaches, such as chemotherapy and radiotherapy, may trigger ROS-mediated cell death mechanisms [57]. Investigating the effects of fucoidan on ROS-mediated ER stress leading to apoptosis and developing a potential therapeutic strategy for the treatment of cancers would be beneficial to cancer patients.

On the other hand, another group of free radical-related materials is reactive nitrogen species (RNS) including nitric oxide (NO) and a series of metabolic compounds from NO. As it known that NO possesses its own unique characteristics, playing important roles in cell signal transduction, anti-infective reaction, as an immune-modulator, etc. It reported that significantly enhanced generation of superoxide and NO from fucoidan treatment in *Leishmania donovani*-infected BALB/c mice in vivo [58], one of the mechanisms is fucoidan can induce Th1 cytokines and NO generation in infected macrophages. However, it is a less report about the NO induction from fucoidan treatment in cancer cells, or any role of fucoidan-induced NO in biological function of cancer cells. In the future, it seems to be an interesting and important study about the effect of fucoidan-induced NO on cancers.

#### 2. Anti-inflammation

Inflammation and immunity play important roles in the development of tumorigenesis [59]. Current epidemiological and preclinical results strongly support an anti-inflammatory approach to treating cancers. Several therapeutic agents targeting cancer-derived inflammatory responses and related signaling molecules, cytokines, transcription factors, and immune cells are being developed and tested [60]. Inflammation, cancer recurrence and cancer metastasis have a complicated relationship. Inflammatory responses play important roles in tumor development, including metastasis [15]. Recently, scientists at Cold Spring Harbor Laboratory and colleagues have demonstrated that sustained lung inflammation can cause dormant breast and prostate cancer cells that have traveled to the lungs to awaken and begin to divide [59]. These cells can now form metastases in the lungs. Specifically, the team showed that chronically sustained lung inflammation, caused either by exposing mice to tobacco smoke or to nasal instillation of a bacterial endotoxin component (lipopolysaccharide, LPS) induced common white blood cells (neutrophils) to awaken nearby dormant cancer cells in an extraordinary way. Interestingly and importantly, fucoidan (from *Sargassum hemiphyllum*) has anti-inflammatory effects by inhibiting LPS-induced inflammatory responses in RAW 264.7 mouse macrophages [61].

Recently, an exploratory human clinical study with a prospective, open-label, single-arm design was conducted in advanced cancer patients to examine the efficacy of fucoidans, with a specific focus on inflammation in relation to patients’ quality of life (QOL) scores [62]. This clinical study revealed that levels of several pro-inflammatory cytokines, such as IL-1β, IL-6, and TNF-α, were significantly reduced after a short period of fucoidan administration (from Mozuku, *C. novae*-*caledoniae* Kylin) in various advanced cancer patients. Interestingly, a subgroup analysis showed that the responsiveness of IL-1β was significantly correlated with the overall survival rate of cancer patients. This responsiveness and relationship might be a useful prognostic biomarker for advanced cancer patients receiving fucoidan. Importantly, this study was the first to establish a close association among fucoidan, cancer, and inflammatory responses and to provide evidence of the anti-inflammatory effects of fucoidan for human advanced cancer patients [62]. This knowledge of treating chronically sustained tissue inflammation may inspire the design of new strategies to prevent cancer recurrence and metastasis.

#### 3. Cell cycle arrest

A cell cycle consists of G0 (quiescence), G1 phase, S phase, G2 phase and M phase; cell cycle progression is controlled by cyclins and CDKs [63]. Some studies have reported effects of different species of fucoidan on cell cycle arrest in different tumor cells. For example, fucoidan induces G1 arrest of cell cycle progression in human bladder cancer EJ cells associated with down-regulation of cyclin D1, cyclin E, and cyclin-dependent-kinases (Cdks) in a concentration-dependent manner [64]. Fucoidan inhibits human colon cancer cell, HT29 growth, induces G1-phase-associated up-regulation of p21WAF1 expression, and suppresses cyclin and cyclin-dependent kinase expression via activation of the Akt signaling pathway [36]. Fucoidan-induced G1 phase arrest is caused by the activity of the p16INK4a-Rb and p14Arf-p53 pathways [33]. It will be important to examine the role of fucoidan in the regulation of cell cycle arrest in anti-cancer effects on different tumor tissues or cells.

#### 4. Anti-angiogenesis

Angiogenesis, a complicated process of new blood vessel formation from pre-existing vasculature, is crucial for malignant tumor growth and tumor metastasis [65]. The process of angiogenesis can be divided into stages of increased vaso-permeability, endothelial cell migration by degradation of the extracellular matrix (ECM), differentiation and new vessel formation, and vessel maturation. Pro-angiogenic molecules, including vascular endothelial growth factor (VEGF), bFGF, IL-8 and PAI-1 [66, 67], play vital roles in angiogenesis. The anti-tumor signaling pathways of fucoidan anti-angiogenesis include inhibition of the VEGF receptor 2 (VEGFR2)/Erk/VEGF signaling pathway [67], down-regulation of MMP-2 activity and VEGF/hypoxia-inducible factor-1 (HIF-1) signaling [68], and reduction of angiogenesis through AKT/MMP-2 signaling pathways by activating JNK and p38 [69], etc.

Many researchers have investigated the influence of different species of fucoidan on angiogenesis-related reactions of different cancer cells. Fucoidan from *Sargassum fusiforme* (FSF) inhibits angiogenesis of human microvascular endothelial cells (HMEC) and micro-vessel formation by lung cancer cell A549 xenografts in nude mice. FSF inhibits VEGFR2/Erk/VEGF signaling pathways in vitro and in vivo in nude mice [67]. FSF also exerts anti-angiogenic effects on the human hepatocellular carcinoma cell lines Bel7402, SMMC7721 and Huh7 [70]. In addition, *S. thunbergii*-derived fucoidan inhibits angiogenesis by downregulating MMP-2 activity and VEGF/hypoxia-inducible factor-1α (HIF-1α) signaling in HUVEC cells and inhibits lung cancer cell A549 migration and proliferation [68]. By contrast, in the presence of basic fibroblast growth factor (FGF-2), fucoidan (from *L. japonica*) induces angiogenesis through AKT/MMP-2 signaling by activating p38 and JNK for in vitro tube formation and cell migration [69]. On the other hand, fucoidan (from *Sargassum integerrimum*, SPS) significantly reduces A549 cell viability and induces cell apoptosis, loss of mitochondrial membrane potential, generation of ROS and G2/M phase cell cycle arrest. SPS also inhibits the proliferation, migration and cord formation of human umbilical vein endothelial cells (HUVECs) in vitro and prevents the vascular development of zebrafish embryos in vivo [71]. These findings have provided insights on the potential pharmacological application of fucoidan as an anti-tumor and antiangiogenic agent against cancers [71].

#### 5. Inhibition of metastasis, migration and invasion

Metastasis is a lethal hallmark of cancer, with most cancer patients dying as a result of the dissemination of the disease to other organs rather than as a consequence of the primary tumor in medical records [72]. Malignant cells migrate (metastasize) from the primary tumor to other sites to develop secondary tumors in cancer patients; these secondary tumors resist conventional treatments and eventually cause failure of vital organs [14]. The tumor microenvironment, which has an insufficient glucose supply, low pH and hypoxia (low oxygen tension), plays a key role in tumor metastasis. The hypoxic environment in tumors is an important factor that causes tumor metastasis by activating hypoxia-inducible factor-1α (HIF-1α). A key event and process in promoting stationary tumor cells at the primary site to migrate and invade is the epithelial-to-mesenchymal transition (EMT) program, which involves down-regulation of E-cadherin expression and up-regulation of N-cadherin. The degradation of the basement membrane, including the ECM, plays an important role in invasion. Metastasis is a complex set of biological processes and involves an orderly sequence of basic steps such as local invasion, intravasation, and survival of cancer cells in circulation, extravasation, migration, colonization and angiogenesis. A simplified summary of metastasis as cell migration (migration) and invasive behavior (invasion) is applied in this review as for angiogenesis, as discussed above.

Metastasis involves complex and redundant signal transduction pathways. The signal transduction pathways relevant to metastasis, migration and invasion involve the tumor cells, and the microenvironment mediates tumor migration at the primary site, survival and arrest in the bloodstream, invasion and progressive outgrowth at distant organs. Many researchers have investigated the influence of fucoidan on anti-metastasis activity in various cancer types [20]. Fucoidan anti-tumor signaling pathways relevant to metastasis migration and invasion involve the expression of matrix metalloproteinases (MMPs) modulated by transcription factors NF-κB, AP-1 and upstream MAPK and phosphoinositide 3-kinase (PI3K)-Akt pathways. Activation of HIF-1α plays an important role in promoting tumor angiogenesis, growth and metastasis. The PI3K/Akt/mammalian target of rapamycin (mTOR) and ERK pathways control the translation of HIF-1α [73], and HIF-1α activates VEGFs, u-PA, and MMP-2 and -9 to promote tumor metastasis.

Fucoidan (from *F. vesiculosus*) alters the EMT process and then reduces metastasis by a ubiquitin-dependent proteasome pathway to degrade transforming growth factor-beta receptors (TGFRs), resulting in EMT-relevant morphological alterations and the expression of EMT-relevant markers in the mouse breast cancer cell line 4T1 and human breast cancer cell lines MDA-MB-231 and MCF-7 in vitro and in vivo [28]. Fucoidan (*F. vesiculosus*) also inhibits the migration of human lung carcinoma CL1-5 cells and mouse Lewis lung carcinoma LLC1 cells by inhibiting the TGF-β/TGFR pathway and down-regulating the downstream FAK signaling pathway in tumor tissues. In addition, fucoidan reduces tumor size in LLC1-xenograft male C57BL/6 mice and decreases tumor growth by modulating the TGFR/Smad7/Smurf2-dependent axis, leading to TGFR protein degradation and inhibition of lung cancer cell progression in vitro and in vivo [26]. Fucoidan (from *F. vesiculosus*) also exerts anti-metastatic effects on human hepatocellular carcinoma cells (HCC) cells: Huh-7, SNU-761, and SNU-308 [74]. Fucoidan inhibits the growth and migration of human bladder cancer cells; specifically, it inhibits cell growth via p21WAF1-mediated G1-phase cell-cycle arrest by activation of AKT. AKT activation has been reported to be involved in the inhibition of migration through the MMP-9/NF-κB/AP-1 pathways [75]. Fucoidan suppresses the invasion of HCC cells (Huh-7, SNU-761) with hepatoprotective effects through up-regulation of p42/44 MAPK (ERK)-dependent NDRG-1/CAP43 under normoxic conditions and partly through up-regulation of p42/44 MAPK-dependent VMP-1 expression [76]. Fucoidan-like polysaccharides (from *Sargassum thunbergii*) inhibit migration and angiogenesis (i.e., tube formation of endothelial cells) and suppress the migration and proliferation of lung cancer cells via reduction of MMP-2 enzymatic activity, expression at the transcriptional level, and downregulation of VEGF and HIF-1α expression [77]. Fucoidan (from *Sargassum hemiphyllum*) decreases the invasion activity of HCC cells (Huh6, Huh7, SK-Hep1 and HepG2 cells) via downregulation of the microRNA-29b-DNMT3B-MTSS1 axis and inhibition of TGF-β signaling [78]. These effects partially result from the inhibition of EMT, i.e., increased E-cadherin and decreased N-cadherin and prevention of extracellular matrix degradation via increased TIMP-1 and decreased MMP-2 and -9 [78]. The PI3K/Akt signaling pathway plays important roles in cancer development processes, such as degradation of the ECM, cell migration, adhesion, and tumor angiogenesis. Fucoidan inhibits the migration of human colon cancer HT-29 cells by decreasing the expression of MMP-2 via the downregulation of PI3K/Akt/mTOR [79]. In hypoxic human bladder cancer cells (T24) cells, fucoidan inhibits hypoxia-stimulated H_2_O_2_ formation, HIF-1α accumulation, transcriptional activity, VEGF secretion, migration and invasion. Fucoidan also downregulates hypoxia-activated phosphorylation of signaling molecules in the PI3K/AKT/mTOR/p70S6K/4EBP-1 pathway in T24 cells. Blocking PI3K/AKT or mTOR activity strongly diminishes hypoxia-induced HIF-1α expression and VEGF secretion [80]. In addition, fucoidan significantly attenuates angiogenesis in vitro and in vivo as evidenced by the reduction of tube formation by hypoxic human umbilical vascular endothelial cells and blood capillary generation in the tumor. Similarly, administration of fucoidan also inhibits HIF-1α and VEGF expression in female athymic nude mice, BALB/c injected with T24 cells in vivo, accompanied by a reduction of tumor growth [80].

The anti-cancer activity and underlying mechanisms of fucoidan (*U. pinnatifida* sporophylls) in human hepatocellular carcinoma SMMC-7721 cells have been investigated. The results showed that fucoidan induces apoptosis via the ROS-mediated mitochondrial pathway by increasing ROS production, depleting intracellular glutathione (GSH)-induced mitochondrial oxidative damage (including a decrease in the number of mitochondria and mitochondrial swelling), MMP depolarization, and release of cytochrome c, combined with downregulation of *XIAP* and *Livin* and activation of caspase-3 and -9 [81]. Fucoidan (*U. pinnatifida*) inhibits the synthesis and secretion of VEGF-C and HGF, cell invasion and lymphatic metastasis in mouse Hca-F hepatocellular carcinoma cells with high lymphatic metastatic activity. Lymphangiogenesis is one of the promoters of tumor lymphatic metastasis. However, the inhibitory effect of fucoidan on lymphangiogenesis remains unclear. Fucoidan also suppresses lymphangiogenesis in CoCl_2_-treated Hca-F cells and in Hca-F-engrafted mice [73]. In addition, accompanied by a reduction in HIF-1α nuclear translocation and activity, fucoidan significantly reduces the levels of phosphorylation of PI3K, Akt, mTOR, and ERK and decreases NF-κB and MMP-2 and -9 while increasing TIMP-1 levels. These results indicate that the anti-metastasis and anti-lymphangiogenesis activities of fucoidan are mediated by suppressing HIF-1α/VEGF-C, which attenuates the PI3K/Akt/mTOR signaling pathways [73]. Fucoidan (*U. pinnatifida*) suppresses the proliferation, migration and tube-like structure formation of human lymphatic endothelial cells (HLECs) and has inhibitory effects on tumor-induced lymphangiogenesis in vitro. Additionally, fucoidan has a dose-dependent depressive effect on the expression of prospero homeobox protein 1 (PROX1), vascular endothelial growth factor receptor 3 (VEGFR3), NF-κB, phospho-PI3K and phospho-Akt in HLECs [82]. Moreover, the anti-lymphangiogenesis effect of fucoidan was assessed by using a mouse tumor model in which male 615 mice were inoculated with Hca-F cells with high lymphatic metastatic activity. Fucoidan inhibits tumor lymphangiogenesis and lymphatic metastasis by suppressing the NF-κB/PI3K/Akt signaling pathway through reduced levels of PROX1 and VEGFR3 in vivo in mice [82]. An understanding of the complex signal transduction of metastatic progression in cancer and the microenvironment is necessary to further apply fucoidan in combination with therapeutic agents to develop new therapeutic strategies to prevent and treat metastasis in cancer patients (see below sections).

#### 6. Immunological reactions

The immune-prevention effects of fucoidan on cancer demonstrate that fucoidan (from *F. vesiculosus*) enhances immune responses in immune cells including natural killer (NK) cells and macrophages. In general, immune-related cells produce various cytokines, e.g., IL-6, TNFα and IFNγ, and free radicals (H_2_O_2_ and NO) that exert tumoricidal activity on tumors or cancer cells.

Fucoidan-mediated macrophages play another important role in the immunological reactions of tumor/cancer. Fucoidan (from *F. vesiculosus*) inhibits tumor cell migration and lymphocytes in tumor microenvironment recruitment by suppressing or downregulating some cytokines and chemokines. For example, the involvement of an “alternatively activated” macrophage (M2)-type chemokine CCL22 (a CC-chemokine subfamily member of a macrophage-derived chemokine) via NF-κB-dependent transcription regulation was reported. Mechanistically, fucoidan inhibits CCL22 by suppressing p65-NF-κB phosphorylation and nuclear translocation. Moreover, p38-MAPK and PI3K-AKT also affect CCL22 expression through differential modulation of NF-κB transcriptional activity. This macrophage-mediated immune process may provide a novel and promising tumor immunotherapy via application of fucoidan [83].

**Fig. 2:**
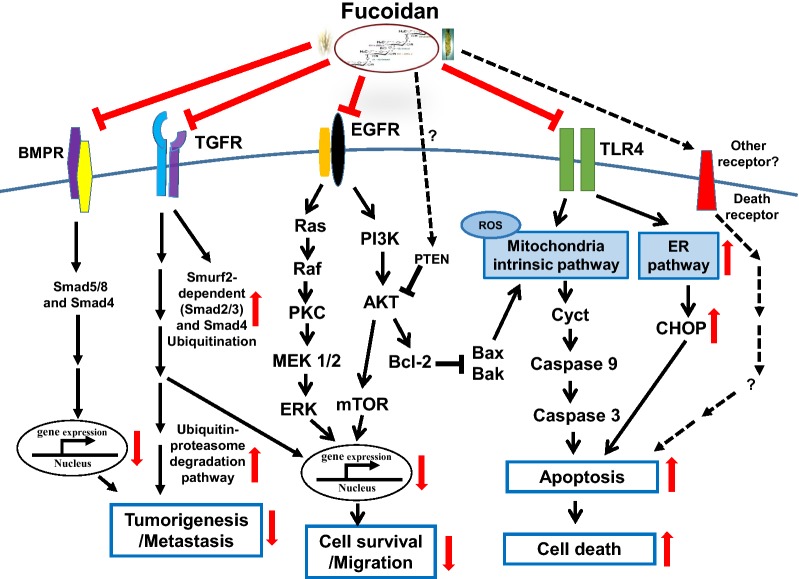
Fucoidan interacts receptors-mediated anti-cancer pathways and reactions

Fucoidan, a kind of polysaccharides derived from various brown seaweeds, exerts different anti-cancer functions. One controversial area of fucoidan anti-cancer activity is the variability in different sources of fucoidan and consequent differences in the anti-cancer activities of fucoidan. There are many “factors” of fucoidan involved in its anti-cancer functions, such as the source of fucoidan, i.e., the species of brown seaweed [53, 54, 84], the effective concentration, structural characteristics, sulfate content, molecular weight, purity, and isolation/extraction methods. The kind of cancer cells tested by investigators can also influence the anti-cancer effects of fucoidan that are observed. Future work further examining and comparing the relationship between structural characteristics and anti-cancer activity for various kinds of fucoidan is needed. Since current results suggest that fucoidan exerts both preventive and therapeutic effects in cancer mouse models (see the sections below and additional papers), fucoidan has been proposed for use as a nutritional/dietary supplement to prevent and inhibit cancer progression. Here we summarize fucoidan-mediated inhibitory mechanisms and reactions on relevant receptors, as in Fig. 2.

### Fucoidan and fucoidan-derived products have been marketed as dietary supplements or nutraceuticals for cancers

Cancer is among the currently leading causes of deaths worldwide, with approximately 1.7 million new cases and more than half a million cancer deaths projected annually in the coming years. Over the next 20 years, the number of new cases is projected to increase by approximately 70% [85]. Current treatments include chemotherapy, radiation, and surgery, however the effects of these procedures may damage not only the tumor tissue but also normal tissue in patients. Weinberg and Hanahan have described a set of six hallmarks of cancer that may help distinguish the characteristics of normal and tumor tissue and perhaps provide better alternative treatments [86]. These hallmarks include sustained proliferative signaling, evasion of growth suppressors, activation of invasion and metastasis, replicative immortality, induction of angiogenesis, and cell death resistance.

Fucoidan has been reported to have advantages of less toxicity and greater oral bioavailability, and the induction of fucoidan-mediated apoptosis and related reactions in various kinds of cancer cells have been discussed above. Fucoidan and fucoidan-derived products have recently been commercialized as dietary supplements or nutraceuticals for various diseases, including cancer [87].

#### Anti-cancer effects of fucoidan combinations as an adjuvant

Several studies have reported that fucoidan and its combinations can induce apoptosis in a variety of cancers. Studies of fucoidan and combinations of fucoidan with clinical drugs in adjuvant settings have suggested that fucoidan may reduce the toxicity of certain anti-cancer drugs. These studies suggest that fucoidan could be used in adjuvant settings for cancer management. Synergistic effects of fucoidan with clinically used drugs in the inhibition of various tumors and/or cancers in mice have been reported, and some examples are discussed below.

Synthetic drugs used as chemotherapeutics agents are frequently limited by their side effects and drug resistance. Fucoidan has been investigated as a dietary supplement or synergetic anti-tumor agent to reduce the toxicity and/or enhance the efficacy of chemotherapeutic drugs. There are many examples of the combination of fucoidan with clinical chemicals or chemotherapeutics agents (i.e., combination therapy). Fucoidan (from *F. evanescens*) combined with cyclophosphamide significantly inhibits metastases in LLC-transplanted C57BL/6 mice in vivo [87]. The decrease in oxidative stress and matrix metalloproteinases due to treatment with fucoidan and vitamin C complex suppress tumor invasion [88]. Fucoidan (from *C. okamuranus*) combined with FOLFOX was used to treat advanced or recurrent colorectal cancer. Patients treated with the combination therapy could receive prolonged chemotherapy time without any signs of fatigue and had a more favorable prognosis, although the survival time of patients was not significantly different from that of the control group [89]. Co-treatment with fucoidan from Mozuku seaweed (*C. novae*-*caledoniae* Kylin) and chemotherapeutic agents, such as tamoxifen, cisplatin and paclitaxel enhance anti-cancer activities such as inhibition of cell growth, apoptosis and cell cycle arrest in human MDA-MB-231 and MCF-7 breast cancer cells [90]. Using an MTS assay [CellTiter 96^®^ AQ_ueous_ One Solution Cell Proliferation Assay (MTS), with solution reagent contains a tetrazolium compound, inner salt; MTS from Promega Corporation, USA] in vitro, fucoidan (from *Saccharina cichorioides*) at a nontoxic dosage combined with resveratrol increases resveratrol-induced apoptosis of HCT116 human colon cancer cells via a caspase-dependent pathway [91]. The anti-cancer effects of fucoidan in combination with the tyrosine kinase inhibitor (TKI) lapatinib have been examined, and the results have shown that this combination has synergistic growth inhibitory effects on the human Caucasian esophageal carcinoma cell line OE33 [92]. Inhibition of lung cancer in vivo and in vitro by fucoidan (from *F. vesiculosus*) has been examined. Positive synergistic cytotoxic effects of fucoidan combined with cisplatin on both LLC1 mouse lung carcinoma cells, as assessed by MTT assays, and mice have been reported. A role of the Smurf2-dependent ubiquitin–proteasome pathway in TGF receptor degradation has been proposed [26]. Fucoidan upregulates TLR4/CHOP-mediated caspase-3 and PARP activation and enhances cisplatin-induced cytotoxicity in human lung cancer cells [25]. Moreover, in human clinical studies, combined cisplatin and fucoidan treatment effectively increased the survival rate of patients with lung cancer in Taiwan. The results indicated that fucoidan exerts a greater anti-tumorigenic effect as an adjuvant, second-line anti-cancer diet supplement and may also regulate immune functions, while cisplatin remains a first-line anti-cancer drug for chemotherapy in clinical human lung cancer treatment [25]. In the future, in vivo and advanced preclinical studies are needed to evaluate the safety and utility of fucoidan combination treatments in lung cancer patients. Moreover, the findings suggest that the combination of fucoidan and cisplatin is a potentially effective therapeutic agent or strategy for preventing human lung tumorigenesis [25]. Additional relevant information about fucoidan combinations as an adjuvant in anti-cancer therapy is provided and explained in the following sections.

### Case studies of fucoidan in complementary therapy and as an alternative medicine in animal and mouse models

Anti-cancer activities of fucoidan in vivo (animal, mouse models) and human clinical trials have been reported. Here, we will discuss some cases and examples of the use of fucoidan as a complementary therapy or food supplement in complementary alternative medicine in the treatment of cancer in animal and mouse models.

#### Zebrafish embryo and zebrafish model in vivo experiments (fucoidan reduces radiotherapy sensitization effects of high-dose radiation of cancer in zebrafish embryos and zebrafish models)

Common side effects of radiotherapy include tissue fibrosis and secondary and primary cancers. Patients with cancer often die not of cancer but of the side effects of radiotherapy. Using zebrafish embryos as a model, the radioprotective effects of fucoidan on embryos were examined. Compared to the control group (embryos exposed to radiation only), the embryo group pretreated with fucoidan before radiation was protected from death or embryonic defects [93]. Moreover, compared to the control group, adult zebrafish fed fucoidan for 1 month prior to exposure to radiation showed reduced expression of apoptosis and fibrosis marker genes. These results again prove the role of fucoidan in preventing radiation-induced fibrosis.

#### Breast cancer in vitro and in vivo mouse model experiments

Fucoidan induces changes in EMT and decreases metastasis by enhancing the ubiquitin-dependent degradation of transforming growth factor-beta receptors (TGFRs) in breast cancer. Fucoidan inhibits the growth of human breast cancer cells such as 4T1 and MDA-MB-231 and decreases their cell colony formation. Moreover, fucoidan reduces metastatic lung nodules in 4T1 xenograft female Balb/c mice. In 4T1 and MDA-MB-231 cells, fucoidan effectively reverses TGFR-induced EMT morphological changes, up-regulates epithelial markers, down-regulates mesenchymal markers, and decreases the expression of the transcriptional repressors Snail, Slug, and Twist [28]. Moreover, fucoidan inhibits migration and invasion during EMT, suggesting the involvement of TGFR-mediated signaling in breast cancer cells. Fucoidan decreases TGFRI and TGFRII proteins and affects downstream signaling molecules, including Smad2/3 phosphorylation and Smad4 expression. To elucidate how fucoidan decreases TGFRI and TGFRII proteins in MDA-MB-231 cells, ubiquitination activity and down-regulation of TGFRs were investigated. Fucoidan enhanced proteasome-mediated degradation/ubiquitination of TGFRs. This study was the first to identify a novel mechanism for fucoidan anti-tumor activity, namely regulation of EMT via modulation of TGFR/Smad-dependent signaling, which leads to an inhibition of breast cancer cell growth in vitro and in vivo. The current findings indicate that fucoidan has potential as a therapeutic intervention for controlling breast cancer or other cancers and that manipulation of the proteasomal ubiquitin-dependent degradation of proteins in relation to the TGFR/Smad/Snail, Slug, and Twist/EMT axes should be a beneficial strategy for cancer patients.

#### Fucoidan inhibits human breast cancer cell growth through dual regulatory mechanisms

Fucoidan inhibits the progression of human breast cancer cells by regulating the microRNA-29C/ADAM12 and microRNA-17-5P/PTEN axes [94]. Fucoidan also inhibits the EMT of human breast cancer cells and further inhibits the survival of cancer cells.

#### Fucoidan inhibition of lung cancer in vitro and in vivo: role of the Smurf2-dependent ubiquitin proteasome pathway in TGF-β receptor degradation and prevention of tumor formation through early consumption of fucoidan

Fucoidan reduces tumor size in LLC1-xenograft male C57BL/6 mice. Moreover, LLC1-bearing mice continuously fed fucoidan showed greater anti-tumor activity than mice fed discontinuously. Fucoidan inhibits the in vitro growth of lung cancer cells. TGFRs play important roles in the regulation of proliferation and progression, and high TGFRI expression in lung cancer specimens is associated with a worse prognosis. In lung cancer cells, fucoidan effectively reduces TGFRI and TGFRII protein levels in vivo and in vitro. Moreover, fucoidan reduces TGFR downstream signaling events, including Akt, Erk1/2, and FAK phosphorylation in the Smad2/3 and non-Smad pathways [26]. Furthermore, fucoidan suppresses lung cancer cell mobility upon TGF-β stimulation. Fucoidan enhances the ubiquitination proteasome pathway (UPP)-mediated degradation of TGFRs in A549 and CL1-5 cells. Mechanistically, fucoidan promotes the conjugation of Smurf2 and Smad7 with TGFRs, resulting in TGFRs degradation; however, Smurf2–shRNA abolishes fucoidan-enhanced UPP-mediated TGFRs degradation. This study was identified a novel mechanism for the anti-tumor activity of fucoidan, namely decreasing tumor growth by modulating the TGFR/Smad7/Smurf2-dependent axis, leading to TGFR protein degradation and inhibition of lung cancer cell progression in vitro and in vivo. Fucoidan inhibits the viability of human NSCLC cells and mouse lung cancer cells, reduces lung tumorigenesis (tumor volume and weight), and inhibits TGFRI/II protein expression in LLC1-xenograft mice orally fed fucoidan. These novel findings suggest that fucoidan enhances the Smurf2-mediated ubiquitination of TGFRs and consequently TGFR degradation. In parallel, fucoidan inhibits TGFR downstream Smad and non-Smad pathways (FAK, Akt and Erk) and suppresses cell mobility. These findings indicate that fucoidan is a potentially promising therapeutic agent or dietary supplement for the prevention of lung tumorigenesis that acts via the Smurf2-dependent ubiquitin degradation of TGF-β receptors.

#### Fucoidan inhibits radiotherapy side effects and improves aberrant fibrosis

Radiotherapy is one of the most important therapies in the treatment of cancer, including lung cancer, breast cancer, and esophageal cancer. In thoracic radiotherapy, most of the radiation is concentrated in the chest cavity, resulting in some side effects, such as radiation pneumonitis and inflammation that gradually leads to fibrosis, including skin, pulmonary and esophageal fibrosis, as well as late cardiac coronary problems and heart failure [95]. Late pulmonary fibrosis may cause a long-term cough, asthma, chest pain, and even inexplicable fever, affecting patients’ quality of life. Fibrosis is pathologically incurable and is irreversible once formed. Even if cancer/tumor is cleared, the site of fibrosis cannot be eliminated. Recent mouse experiments revealed that fucoidan can change the inflammatory response reaction in terms of protein expression, thereby alleviating radiation-induced pneumonitis and fibrosis [95]. An improvement of lung fibrosis was also observed. Unexpectedly, to some degree, fucoidan slows down the fibrosis of internal cells, including those of the heart and coronary arteries. Thus, fucoidan has great potential for application in the treatment or alleviation of pneumonitis and of aberrant fibrosis during radiotherapy of cancer patients.

#### Fucoidan induction of Toll-like receptor 4-regulated reactive oxygen species and promotion of endoplasmic reticulum stress-mediated apoptosis in lung cancer in vitro and in vivo: a potential therapeutic agent for treating lung cancer

Fucoidan effectively inhibits the proliferation of lung cancer cells via the induction of G1 phase arrest and promotes cancer cell death via apoptosis. Moreover, continuous oral feeding with fucoidan significantly prevents tumorigenesis and reduces tumor size in addition to inducing ATF4 and CHOP protein expression in LLC1-bearing mice in vivo. However, the effect and mechanism of fucoidan-induced apoptosis via endoplasmic reticulum (ER) stress are unclear. Fucoidan prevents tumorigenesis and reduces tumor size in LLC1-xenograft male C57BL/6 mice. Fucoidan induces an ER stress response via activating the PERK-ATF4-CHOP pathway, resulting in apoptotic cell death in vitro and in vivo [48]. Furthermore, ATF4-knockdown abolishes fucoidan-induced CHOP expression and rescues cell viability. Specifically, fucoidan increases the intracellular reactive oxygen species (ROS) that are responsible for the increases in ATF4 and CHOP protein expression and activates UPP to induce lung cancer cell death. The ROS scavenger *N*-acetyl-l-cysteine (NAC) abolishes fucoidan-induced ROS and inhibits fucoidan-induced ER stress. These results indicate that ROS generation is involved in fucoidan-induced ER stress-mediated apoptosis. Moreover, Toll-like receptor 4 (TLR4) knockdown attenuates fucoidan-induced ROS and CHOP expression. This indicates a novel mechanism for the anti-tumor activity of fucoidan, namely inhibiting tumor viability by activating the TLR4/ROS/ER stress axis and the downstream PERK-ATF4-CHOP pathway (i.e., via the TLR4/ROS/PERK/eIF2α axes) [48], leading to apoptosis and suppression of lung cancer cell progression in vitro and in vivo. Moreover, TLR4 plays an important role in fucoidan-induced ROS-mediated ER stress and apoptosis. In addition, continuous oral feeding of mice with fucoidan significantly prevents tumorigenesis and reduces tumor size in an LLC1-bearing mouse model and induces ATF4 and CHOP protein expression in mice in vivo. Taken together, these results indicate that fucoidan is a potential preventive and therapeutic agent for lung cancer that acts via activation of the ROS-dependent ER stress pathway.

#### Fucoidan elevation of microRNA-29b to regulate DNMT3B-MTSS1 axis and inhibition of EMT in human hepatocellular carcinoma cells

Accumulating evidence has revealed that fucoidan exhibits anti-tumor activities by arresting the cell cycle and inducing apoptosis in many types of cancer cells, including human hepatocellular carcinoma cells (HCC). Exploring fucoidan effect on microRNA (miRNA) expression, it found that fucoidan markedly upregulates miRNA-29b in human HCC cells. The induction of miRNA-29b is accompanied by suppression of its downstream target DNA methyltransferase 3B (DNMT3B) in a dose-dependent manner [78]. Consistent with these findings, the miRNA and protein levels of metastasis suppressor 1 (MTSS1), a target silenced by DNMT3B, are increased after fucoidan treatment. Furthermore, fucoidan also down-regulates TGFRs and Smad signaling in HCC cells. All of these effects lead to an alteration of EMT (i.e., increased E-cadherin and decreased N-cadherin) and prevention of ECM degradation (via increased TIMP-1 and decreased MMP-2, -9), thus diminishing the invasion activity of HCC cells. The results demonstrate the profound effect of fucoidan not only on the regulation of the miRNA-29b-DNMT3B-MTSS1 axis but also on the inhibition of TGF-β signaling in HCC cells, suggesting the potential of using fucoidan as an integrative therapeutic against HCC invasion and metastasis.

#### Fucoidan prevents cancer cell growth and tumor proliferation in colon cancer-bearing mice: fucoidan decreases IL-6 and CCL2 production and cooperates with p53 to suppress ATM signaling and human colon cancer progression in xenograft-bearing mice

To date, it is unclear whether fucoidan cooperates with the tumor suppressor p53 to further prevent tumor progression and change the tumor microenvironment. Fucoidan collaborates with p53 to prevent spontaneous or etoposide-induced DNA breaks and to regulate the DNA damage response and cell cycle checkpoint. In addition to effectively reducing the side effects and enhancing the therapeutic effects of etoposide, fucoidan prevents tumor development in mice bearing HCT116 human colon cancer cells and suppresses M2 macrophage polarization [96]. Fucoidan supplementation increases cancer cell death and attenuates the adverse effects of etoposide that decrease the production of the pro-inflammatory cytokine IL-6 and chemokine CCL2/MCP-1. Importantly, fucoidan decreases tumor-promoting M2 macrophages in the microenvironment and cooperates with p53 and etoposide to prevent HCT116 tumorigenicity. These results indicate that fucoidan is a promising supplement for the treatment of cancer by enhancing tumor suppressor activity, minimizing the side effects of etoposide chemotherapy, modulating cytokine profiles and altering the tumor microenvironment.

#### Fucoidan exerts multi-functions for reducing chemotherapy side effects: the combination of fucoidan with drugs ameliorates tumor and chemotherapy-induced muscle atrophy in a bladder cancer-bearing mouse model

Clinical chemotherapy drugs used in the treatment of cancer not only kill cancer cells but also potentially destroy normal cells, resulting in an inability to metabolize and absorb nutrients in cancer patients. This loss of nutrients, in turn, leads to weight loss, muscle atrophy, anorexia, digestive system dysfunction, and other health problems in cancer patients. The accumulated malnutrition eventually causes a failure to continue treatment in cancer patients. The diverse reactions induced by malnutrition are called cancer cachexia in medicine. Anti-inflammatory response effects of fucoidan have been observed, and a significant improvement of chemotherapy-induced side effects has been proposed. In experiments with mice with malignant bladder cancer, individual differences were observed among mice receiving chemotherapy or chemotherapy with fucoidan. In brief, fucoidan reduced chemotherapy side effects in three ways, by increasing muscle protein production, reducing muscle atrophy and protecting the metabolism and absorption functions of the gastrointestinal tract [97]. Hence, animal experiments have confirmed that fucoidan significantly improves chemotherapy-induced weight loss, muscle atrophy, and digestive tract dysfunction, in addition to promoting muscle tissue growth and a near-return to the state prior to chemotherapy [97].

#### Fucoidan exerts health promotion and anti-fatigue activity in mice: fucoidan supplementation improves exercise performance and exhibits anti-fatigue actions in a mouse model

Fatigue reduces various resources of cancer patients, affects their nutritional status, increases morbidity and negatively impacts the dose intensity of cancer therapy [25]. In a mouse-exercise model, long-term oral *L. japonica*-derived fucoidan (0.31 g/kg) improved exercise performance and exhibited anti-fatigue activity [98]. Interestingly, the reduced fatigue was associated with decreased plasma lactate, triglyceride and ammonia levels and increased serum glucose [98]. The unique anti-fatigue activity and health promotion effects of fucoidan will be discussed in the next section on fucoidan human clinical trials.

### Case of pharmacokinetics studies of fucoidan in animal models

Pharmacokinetics (PK), a branch of pharmacology investigates the time course of absorption, distribution, metabolism and excretion of drugs from biological system administered to a living organism. At present, the PK study of drugs from original pharmaceutical drug of interest expands to any xenobiotic/chemical, such as herb medicines (e.g., some Chinese medicines: Danshen (*Salvia miltiorrhiza*), Kang-lai-te (*Coix lacryma*), and *Ginkgo bilboa* have been evaluated in randomized controlled clinical trials), food additives, cosmetics, pesticides, etc. By contrast, now, there is only a few studies addressing the absorption, distribution, metabolism (metabolites) and elimination/excretion of fucoidan, which related to pharmacokinetics-like study of fucoidan in animals. Since polysaccharides are not considered to be absorbed orally, it is difficult to understand how systemic effects occur.

In the study of intestinal absorption of fucoidan (from *C. okamuranus*), it observed that oral administration of fucoidan is preferentially accumulated in the liver, accompanied by low systemic blood levels in in vivo experiments of Wistar rats [99]. Under immune-histochemical staining of the small intestines of tested rats, it revealed that fucoidan accumulated in epithelial cells, mononuclear cells and cells in the liver. However, the uptake of fucoidan through the intestinal tract seemed to be low, it was suggested that fucoidan is incorporated into intestinal macrophages. Considering the heterogeneous molecular weight and anionic characteristics of sulfated polysaccharides between heparin and fucoidan, there may exist in some common of their biological functions. It is hypothetical that pharmacokinetics-like of fucoidan may resemble those of heparin does. Therefore, it was proposed that the tested fucoidan in in vivo Wistar rat model could be rapidly eliminated from the serum after its oral administration according to first-order kinetics, leading to low serum fucoidan levels [99].

In addition, the experiments to evaluate the pharmacokinetics and tissue distribution of fucoidan (from *F. vesiculosus*) in rats after a single-dose oral administration were conducted [100]. It found that the tissue distribution of fucoidan after intragastric administration to the rats was characterized, and it exhibited considerable heterogeneity [100]. Specifically, fucoidan preferentially accumulates in the kidneys (AUC_0−t_ = 10.74 µg h/g; C_max_ = 1.23 µg/g after 5 h; AUC, the area under the curve, represents the total drug exposure integrated over time and is an important parameter for pharmacokinetic analyses), spleen (AUC_0−t_ = 6.89 µg h/g; C_max_ = 0.78 µg/g after 3 h), and liver (AUC_0−t_ = 3.26 µg h/g; C_max_ = 0.53 µg/g after 2 h), and shows a relatively long absorption time and extended circulation in the blood, with a mean residence time (MRT) = 6.79 h [100].

As it known pharmacokinetics of drug has emerged as an integral part of a new drug development, especially, when researchers identifying the biological properties of a developing drug. In the future, to follow the rule of PK study of drug, and to further investigate the function of fucoidan in anti-cancer/anti-tumor of human patients, we think that it will be an important project to conduct the experiments of pharmacokinetic studies of fucoidan in cancer cells, cancer-bearing mice (rat), and humans. Indeed, it is necessary to obtain human pharmacokinetic information for fucoidan and to ensure the appropriate use of fucoidan as medicines in the cancer researches.

### Case studies of fucoidan in complementary therapy as alternative medicine: human clinical trials (fucoidan as complementary therapy or a food supplement in the field of complementary alternative medicine)

A variety of studies indicate that fucoidan is unlikely to be used as an individual agent alone for cancer treatment. Here, we discuss research on the potential for orally delivered fucoidan to be used as an adjunct and/or complementary therapy with conventional anti-cancer treatments. Fucoidan may play a role in reducing side effects and in enhancing the therapeutic effects of conventional anti-cancer therapies.

#### A human clinical trial of co-administration of fucoidan and anti-cancer drugs in breast cancer patients

Although many reports of fucoidan or fucoidan-products have demonstrated clear anti-cancer activity in various cancer cells and/or xenograft tumor mouse models, few human clinical studies have investigated fucoidan. Such studies are necessary to examine the effects of fucoidan on human cancer patients. To reassure patients and physicians, any fucoidan application proposed as an adjunct to chemotherapy should not interfere with the serum pharmacokinetics of chemotherapy drugs [101]. In Australia, a study investigated the effect of co-administration of *U. pinnatifida*-derived fucoidan on patients with breast cancer treated with hormonal therapies, letrozole, and tamoxifen. Co-administration of fucoidan resulted in no significant changes in steady-state plasma concentrations of letrozole, tamoxifen, or tamoxifen-derived metabolites or in the risk of clinically significant interactions and adverse effects of fucoidan treatment during the period of study [101].

#### A human clinical trial of fucoidan with anti-cancer drugs showed enhancement of the disease control rate in human patients with metastatic colorectal cancer

A prospective, randomized, double-blind, controlled clinical trial in human patients with metastatic colorectal cancer (mCRC) was conducted between 2014 and 2016 in the Southern city of Taiwan. These patients were treated with folinic acid, 5-fluorouracil, and irinotecan (FOLFIRI) plus bevacizumab (Avastin^®^ Injection) therapy biweekly as the first-line chemo-target regimen. In the study group, each patient received fucoidan powder BID (*bis in die*, twice a day in Latin). In the trial, 60 eligible patients with mCRC were included; 54 patients were enrolled at the end of the trial, including 28 patients in the study group and 26 patients in the control group. Taiwan oligo fucoidan^®^ effectively enhanced the disease control rate (DCR) of mCRC patients [102]. Specifically, at a median follow-up period of 11.5 months, the DCR, defined as the sum of the complete response (CR), partial response (PR) and stable disease (SD) rates, was significantly statistical higher (by 23.6%) in the study group than in the control group (i.e., 92.8% vs 69.2%; p = 0.026). Adverse effects (AEs), the overall response rate (ORR), overall survival (OS), progression-free survival (PFS), and quality of life (QOL) did not differ significantly between the two groups. Moreover, using the Kaplan–Meier method with differences compared using the log-rank test, both the cumulative OS rates and cumulative PFS rates of the study and control groups were examined, and no significant difference was observed between the two groups. Interestingly, trends of improved OS and PFS were also noted in further analyses. Additional studies with a larger sample size were suggested to evaluate whether fucoidan eventually improves OS and PFS in mCRC patients [102]. This study was the first randomized, double-blind, controlled trial evaluating the efficacy of fucoidan as a supplemental therapy in patients with mCRC. The results demonstrated the advantages of fucoidan in improving the disease control rate (DCR) in mCRC patients and provide insights on the development of cancer treatments, particularly the combination of natural or herbal products including fucoidan with chemotherapeutic agents, such as FOLFIRI and/or bevacizumab.

Adopting the concept and approach of precision medicine, another study selected different colorectal cancer cell lines for experiments. Combined use with fucoidan produced better results than chemotherapy drugs (5-FU, Cetuximab, Erbitux^®^, and Avastin^®^) used alone. The study also found that fucoidan increased the synergetic effects of chemotherapy drugs [103]. Based on the results of preliminary experiments in colorectal cancer cells, fucoidan exerts certain adjuvant effects on commonly used chemotherapy drugs. In addition, cell experiments have also shown that the combined use of fucoidan with chemotherapy drugs (5-FU, Cetuximab, Erbitux^®^, and Avastin^®^) produces synergetic effects on controlling the metastasis of cancer cells [103].

#### Human clinical observations and trials of fucoidan combined with the therapeutic agent cisplatin to increase the lifespan of patients with lung cancer

Applying cisplatin with fucoidan was reported to increase the lifespan of patients with lung cancer in Taiwan [25]. The clinical results suggested that the combination of cisplatin and fucoidan exerts a greater anti-tumorigenic effect in patients with lung cancer in Taiwan. Basic research investigated whether fucoidan in combination with the therapeutic agent cisplatin can overcome lung cancer. This novel combination synergistically suppressed lung tumorigenesis in lung cancer cells and LLC1-bearing mice. Moreover, the simultaneous fucoidan and cisplatin treatment enhanced cytotoxicity, inhibited cell viability and induced apoptosis in human lung cancer cells. Specifically, the apoptosis induced by simultaneous treatment with fucoidan and cisplatin in human lung cancer cells partially resulted from the upregulation of caspase-3 and PARP expression (activation) by fucoidan to enhance cisplatin-induced cytotoxicity. Therefore, the current findings suggest that the combination of fucoidan and cisplatin is a potential therapeutic agent for preventing lung tumorigenesis [25].

A preclinical-like study did not analyze whether fucoidan protects patients from cisplatin toxicity, including reducing clinical indicators of toxicity and fatigue. A previous study revealed that fucoidan reduced the occurrence of general fatigue in cancer patients during chemotherapy [89]. In that study, patients who received fucoidan seemed to be able to endure prolonged chemotherapy without fatigue. Moreover, fucoidan may enable the continuous administration of cisplatin for patients with advanced lung cancer and, as a result, prolong the prognosis of such patients [89]. Future randomized, double-blind clinical studies are recommended to investigate whether fucoidan might help cancer-related fatigue as measured by various scales of fatigue, vitality, and well-being and evaluations of quality of life (QOL). Fucoidan is anticipated to improve human health and has been widely distributed as a foodstuff and supplemental agent but not as a drug. However, the detailed mechanism of action of fucoidan and/or fucoidan combined with cisplatin remains to be verified, and its effects in humans have not been determined.

#### A human clinical trial examining the effect of fucoidan anti-inflammatory functions: an exploratory study of the anti-inflammatory effects of fucoidan in relation to quality of life in human patients with advanced cancers

Recently, an exploratory human clinical study was performed. In brief, a prospective, open-label, single-arm clinical design was adopted for advanced cancer patients (28 cancer patients were initially recruited in collaboration with 4 clinics in Japan; at the end of the clinical study, 20 patients who met all the inclusion criteria were analyzed) to examine the efficacy of fucoidans (from Mozuku, *C. novae*-*caledoniae* Kylin), with a particular focus on inflammation in relation to patients’ quality of life (QOL) scores [62]. This clinical study revealed that the levels of some pro-inflammatory cytokines such as interleukin-1β (IL-1β), IL-6, and tumor necrosis factor-α (TNF-α) were reduced after a short period (after 2 weeks) of fucoidan administration in patients with various advanced cancers. Moreover, a subgroup analysis showed that the responsiveness of IL-1β was correlated with the overall survival rate of cancer patients. In contrast to expectations and a previous randomized clinical trial showing that fucoidan could alleviate chemotherapy (Cx)-induced fatigue in patients with colorectal cancer [89], in the study in Japan, QOL scores, including fatigue, were nearly stable and did not improve. Such discrepant results are likely a result of differences in individual patient status in fatigue development and/or the type of questionnaire used [62]. This responsiveness and relationship might be a useful prognostic biomarker for advanced cancer patients receiving fucoidan. Importantly, this study was the first to establish a close association among cancer, inflammatory responses, and fucoidan and to provide evidence of the anti-inflammatory effects of fucoidan in human advanced cancer patients [62]. In the future, larger controlled trials are required to examine the efficacy of fucoidan for advanced cancer patients as complementary medicine and supportive care to alleviate the side effects of chemotherapy.

Whether obtained in basic in vitro or in vivo research studies or human clinical trials, the above research results have proven that fucoidan can exert obvious adjuvant treatment effects on various cancers. These findings directly allow molecular mechanisms in cancer research to be applied in adjuvant treatments, in line with the pursuit of translational medicine and the mindset of establishing a direct link between basic medical research and clinical treatment.

#### An oral administration study of fucoidan to investigate significant factors influencing the absorption of fucoidan

Recently, an oral administration and absorption study of Mozuku fucoidan in 396 Japanese volunteers was performed. Using multiple regression analyses, the significant factors influencing the absorption of fucoidan in the tested urine samples were investigated [104]. The results showed that fucoidan absorption in humans is extremely low; the fucoidan concentration after oral administration was approximately ten times higher in urine than in serum, confirming the intestinal absorption of Mozuku fucoidan in humans. The results indicated that volunteers living in Okinawa prefecture have the maximum value of urinary fucoidan, significantly higher estimated urinary excretion of fucoidan by place of residence, and significantly higher Mozuku fucoidan consumption habits compared with those living outside Okinawa prefecture. In addition, multiple regression analyses showed that Okinawa prefecture as a place of residence was a main significant factor contributing to the estimated urinary excretion of fucoidan in the study. The dietary habit of eating Mozuku fucoidan was suggested to be one of the factors associated with fucoidan absorption [104]. However, the biological mechanisms of fucoidan absorption across the intestinal tract need to be further investigated.

The above results show that fucoidan, whether through basic in vitro to in vivo research studies or clinical trials in humans, has been proven to produce effect of adjuvant therapy on cancer treatment. This allows molecular mechanisms in cancer research to be applied in adjuvant treatment, in line with the pursuit of translational medicine and the mindset of establishing a direct link between basic medical research and clinical application.

In conclusion, understanding the mechanisms underlying the anti-cancer effects of fucoidan, the advantages of combining fucoidan with therapeutic agents in the treatment of cancers, and the pharmacological limitations of fucoidan will aid the development of more informed approaches to treating cancers and may improve current clinical outcomes for cancer patients.

## Abbreviations

PI3K: phosphoinositide 3-kinase
ASK1: apoptosis signal-regulating kinase
mTOR: mammalian target of rapamycin
PARP: poly-ADP-ribose polymerase
ER: endoplasmic reticulum
NAC: *N*-acetyl-l-cysteine
ROS: reactive oxygen species
HIF-1α: hypoxia-inducible factor-1α
EMT: epithelial-to-mesenchymal transition
ECM: extracellular matrix
MMPs: matrix metalloproteinases
VEGF: vascular endothelial growth factor
TGFRs: transforming growth factor receptors
UPP: ubiquitination proteasome pathway
TLR4: toll-like receptor 4
QOL: quality of life
